# Uptake, engagement, and adherence to pre-exposure prophylaxis offered after population HIV testing in rural Kenya and Uganda: 72-week interim analysis of observational data from the SEARCH study

**DOI:** 10.1016/S2352-3018(19)30433-3

**Published:** 2020-02-19

**Authors:** Catherine A Koss, Edwin D Charlebois, James Ayieko, Dalsone Kwarisiima, Jane Kabami, Laura B Balzer, Mucunguzi Atukunda, Florence Mwangwa, James Peng, Yusuf Mwinike, Asiphas Owaraganise, Gabriel Chamie, Vivek Jain, Norton Sang, Winter Olilo, Lillian B Brown, Carina Marquez, Kevin Zhang, Theodore D Ruel, Carol S Camlin, James F Rooney, Douglas Black, Tamara D Clark, Monica Gandhi, Craig R Cohen, Elizabeth A Bukusi, Maya L Petersen, Moses R Kamya, Diane V Havlir

**Affiliations:** Division of HIV, Infectious Diseases and Global Medicine, University of California San Francisco, San Francisco, CA, USA; Division of Prevention Sciences, Department of Medicine, University of California San Francisco, San Francisco, CA, USA; Centre for Microbiology Research, Kenya Medical Research Institute, Nairobi, Kenya; Infectious Diseases Research Collaboration, Kampala, Uganda; Infectious Diseases Research Collaboration, Kampala, Uganda; Department of Biostatistics and Epidemiology, University of Massachusetts Amherst, Amherst, MA, USA; Infectious Diseases Research Collaboration, Kampala, Uganda; Infectious Diseases Research Collaboration, Kampala, Uganda; Division of HIV, Infectious Diseases and Global Medicine, University of California San Francisco, San Francisco, CA, USA; Infectious Diseases Research Collaboration, Kampala, Uganda; Infectious Diseases Research Collaboration, Kampala, Uganda; Division of HIV, Infectious Diseases and Global Medicine, University of California San Francisco, San Francisco, CA, USA; Division of HIV, Infectious Diseases and Global Medicine, University of California San Francisco, San Francisco, CA, USA; Centre for Microbiology Research, Kenya Medical Research Institute, Nairobi, Kenya; Centre for Microbiology Research, Kenya Medical Research Institute, Nairobi, Kenya; Division of HIV, Infectious Diseases and Global Medicine, University of California San Francisco, San Francisco, CA, USA; Division of HIV, Infectious Diseases and Global Medicine, University of California San Francisco, San Francisco, CA, USA; Division of HIV, Infectious Diseases and Global Medicine, University of California San Francisco, San Francisco, CA, USA; Department of Pediatrics, University of California San Francisco, San Francisco, CA, USA; Department of Obstetrics, Gynecology and Reproductive Sciences, University of California San Francisco, San Francisco, CA, USA; Gilead Sciences, Foster City, CA, USA; Division of HIV, Infectious Diseases and Global Medicine, University of California San Francisco, San Francisco, CA, USA; Division of HIV, Infectious Diseases and Global Medicine, University of California San Francisco, San Francisco, CA, USA; Division of HIV, Infectious Diseases and Global Medicine, University of California San Francisco, San Francisco, CA, USA; Department of Obstetrics, Gynecology and Reproductive Sciences, University of California San Francisco, San Francisco, CA, USA; Department of Obstetrics, Gynecology and Reproductive Sciences, University of California San Francisco, San Francisco, CA, USA; Centre for Microbiology Research, Kenya Medical Research Institute, Nairobi, Kenya; Graduate Group in Biostatistics, School of Public Health, University of California Berkeley, Berkeley, CA, USA; Infectious Diseases Research Collaboration, Kampala, Uganda; School of Medicine, Makerere University College of Health Sciences, Kampala, Uganda; Division of HIV, Infectious Diseases and Global Medicine, University of California San Francisco, San Francisco, CA, USA

## Abstract

**Background:**

Optimal strategies for pre-exposure prophylaxis (PrEP) engagement in generalised HIV epidemics are unknown. We aimed to assess PrEP uptake and engagement after population-level HIV testing and universal PrEP access to characterise gaps in the PrEP cascade in rural Kenya and Uganda.

**Methods:**

We did a 72-week interim analysis of observational data from the ongoing SEARCH (Sustainable East Africa Research in Community Health) study. Following community sensitisation and PrEP education, we did HIV testing and offered PrEP at health fairs and facilities in 16 rural communities in western Kenya, eastern Uganda, and western Uganda. We provided enhanced PrEP counselling to individuals 15 years and older who were assessed as having an elevated HIV risk on the basis of serodifferent partnership or empirical risk score, or who otherwise self-identified as being at high risk but were not in serodifferent partnerships or identified by the risk score. PrEP follow-up visits were done at facilities, homes, or community locations. We assessed PrEP uptake within 90 days of HIV testing, programme engagement (follow-up visit attendance at week 4, week 12, and every 12 weeks thereafter), refills, self-reported adherence up to 72 weeks, and concentrations of tenofovir in hair samples from individuals reporting HIV risk and adherence during follow-up, and analysed factors associated with uptake and adherence. This study is registered with ClinicalTrials.gov, NCT01864603.

**Findings:**

Between June 6, 2016, and June 23, 2017, 70 379 community residents 15 years or older who had not previously been diagnosed with HIV were tested during population-level HIV testing. Of these individuals, 69 121 tested HIV-negative, 12 935 of whom had elevated HIV risk (1353 [10%] serodifferent partnership, 6938 [54%] risk score, 4644 [36%] otherwise self-identified risk). 3489 (27%) initiated PrEP, 2865 (82%) of whom did so on the same day as HIV testing and 1733 (50%) of whom were men. PrEP uptake was lower among individuals aged 15–24 years (adjusted odds ratio 0·55, 95% CI 0·45–0·68) and mobile individuals (0·61, 0·41–0·91). At week 4, among 3466 individuals who initiated PrEP and did not withdraw or die before the first visit, 2215 (64%) were engaged in the programme, 1701 (49%) received medication refills, and 1388 (40%) self-reported adherence. At week 72, 1832 (56%) of 3274 were engaged, 1070 (33%) received a refill, and 900 (27%) self-reported adherence. Among participants reporting HIV risk at weeks 4–72, refills (89–93%) and self-reported adherence (70–76%) were high. Among sampled participants self-reporting adherence at week 24, the proportion with tenofovir concentrations in the hair reflecting at least four doses taken per week was 66%, and reflecting seven doses per week was 44%. Participants who stopped PrEP accepted HIV testing at 4274 (83%) of 5140 subsequent visits; half of these participants later restarted PrEP. 29 participants of 3489 who initiated PrEP had serious adverse events, including seven deaths. Five adverse events (all grade 3) were assessed as being possibly related to the study drug.

**Interpretation:**

During population-level HIV testing, inclusive risk assessment (combining serodifferent partnership, an empirical risk score, and self-identification of HIV risk) was feasible and identified individuals who could benefit from PrEP. The biggest gap in the PrEP cascade was PrEP uptake, particularly for young and mobile individuals. Participants who initiated PrEP and had perceived HIV risk during follow-up reported taking PrEP, but one-third had drug concentrations consistent with poor adherence, highlighting the need for novel approaches and long-acting formulations as PrEP roll-out expands.

**Funding:**

National Institutes of Health, President’s Emergency Plan for AIDS Relief, Bill & Melinda Gates Foundation, and Gilead Sciences.

## Introduction

Despite remarkable progress in scaling up HIV testing and treatment worldwide, 1·7 million new HIV infections occurred in 2018, signalling what UNAIDS has termed a prevention crisis.^1^ Oral pre-exposure prophylaxis (PrEP) is highly effective^2^ and has the potential to accelerate reductions in HIV incidence as part of combination approaches to HIV prevention. Reduced HIV incidence has been observed in high-income populations of men who have sex with men for whom antiretroviral therapy (ART) and PrEP have been scaled-up.^3,4^ PrEP is recommended in global guidelines^5^ and is being introduced across southern and eastern Africa, where the burden of new infections is the greatest.

In generalised epidemic settings, strategies are needed to identify and engage individuals who might benefit from HIV prevention services, including PrEP. Most studies in sub-Saharan Africa have offered PrEP to prespecified populations who are at high risk, such as young women,^6^ sex workers,^7^ and serodifferent couples,^8^ who either present to clinical care or are selectively recruited from the community. Comprehensive HIV testing and universal access to PrEP for all people without HIV who are at high risk of infection presents an opportunity to offer PrEP to people who might not otherwise engage in health systems, and to assess PrEP engagement at a population level.

We offered PrEP during population-level HIV testing in an ongoing study in rural Kenya and Uganda, using an inclusive approach to PrEP eligibility, with options for same-day or rapid PrEP start and community-based or facility-based service delivery. We aimed to estimate the proportion of the entire population at elevated HIV risk to establish a denominator for calculations of population-level PrEP uptake; assess programme engagement, medication refill, and adherence (measured by self-report and objective metrics); and identify gaps in the PrEP cascade.

## Methods

### Study design and participants

In this analysis, we report interim observational data from an ongoing study of a PrEP intervention in 16 communities in three regions of eastern Africa with varying HIV prevalence among individuals aged 15 years or older: western Kenya (19%), eastern Uganda (4%), and western Uganda (7%).^9^ As part of the second phase of the Sustainable East Africa Research in Community Health (SEARCH) study, we did population-level HIV testing and implemented a PrEP intervention^10^ before national PrEP roll-out in Uganda and Kenya. The study population included adults (aged ≥15 years) in the SEARCH study. Here, we report an interim analysis up to 72 weeks of follow-up to address knowledge gaps in the population-level PrEP cascade in the context of roll-out and scale-up in the region.

Ethical approval for the study was by the institutional review boards of Makerere University (Kampala, Uganda), Kenya Medical Research Institute (Nairobi, Kenya), and University of California San Francisco (San Francisco, CA, USA). All participants provided verbal consent; PrEP participants provided written informed consent in their preferred language.

### Procedures

We did population-level HIV and multi-disease testing using a hybrid mobile testing approach.^11^ We held health fairs at multiple locations across each community over 2 weeks, followed by home-based testing for non-attendees. 1 month before testing events, we conducted community mobilisation and sensitisation on PrEP in collaboration with health officials and government leaders. Study staff disseminated information on PrEP at local events and meetings, including with political and religious leaders; health workers; patients with HIV; adolescents and young adults; parents; and fishing, bar, and transport workers (occupations associated with higher HIV prevalence or transactional sex).^12,13^

Community members received basic group-based education on PrEP upon arrival at health fairs, then attended stations for collection of sociodemographic information, HIV testing, and post-test counselling. During post-test counselling, we offered enhanced individual PrEP counselling to people who tested negative for HIV and had an elevated risk of HIV acquisition: individuals in serodifferent partnerships; individuals classified as being at risk on the basis of an empirical HIV risk-prediction algorithm (risk score); or individuals who were neither in serodifferent partnerships nor identified by risk score, but who otherwise self-identified as being at risk. Serodifferent partnerships were self-identified male-female spouses of differing HIV status or individuals who self-reported having a partner with HIV. As previously described,^10,14^ the risk score was developed by applying ensemble machine learning to HIV seroconversion and sociodemographic data from the first 2 years of the SEARCH test-and-treat trial^9^ (appendix p 1). Data on sexual behaviour were not previously collected and were therefore not available for risk-score development. Among individuals not known to be I n serodifferent partnerships, the score analysed region and socio-demographicf characteristics (age, sex, marital status, polygamy, education, occupation, alcohol consumption, and circumcision) entered in real-time into tablet computers at health fairs before the HIV-testing station. Health-fair staff recorded a dichotomous output (at risk or not at risk) on a paper card for the participant to give to the HIV post-test counsellor.

Post-test counsellors discussed individuals’ potential HIV risk (eg, knowledge of partner status, concurrent partnerships, condom use, circumcision). Individuals assessed to be at an elevated HIV risk (based on serodifferent partnership, risk score, or otherwise self-identified risk) received enhanced counselling on PrEP, including information on who might benefit, how PrEP is taken, and potential adverse effects. We offered same-day or rapid PrEP initiation through SEARCH at local government HIV clinics (with one-time, study-provided transport). In 14 of 16 communities, on-site PrEP start was also offered at SEARCH health fairs. For individuals who received home-based testing, one study staff member did all of the described steps, including provision of information about PrEP, risk-score assessment, HIV tesing and post-test counselling, and offer of PrEP through local clinics (but not on-site at home).

During population-level testing, we measured HIV-1 RNA among individuals with HIV. We assessed viral suppression (≤500 RNA copies per mL) among linked HIV-positive spouses in serodifferent partnerships, but real-time results were not available for PrEP counselling. After population-level testing, we offered ongoing PrEP initiation through SEARCH at local clinics, where we did intensive outreach. Patients with HIV were asked to bring their partners who were HIV-negative or with unknown HIV status to the clinic for HIV testing and the offer of PrEP initiation.

PrEP eligibility criteria included negative HIV testing within 4 weeks, no known hepatitis B infection, and no acute HIV symptoms. Baseline creatinine testing was done but PrEP was provided at enrolment before the receipt of laboratory results. After providing written informed consent, participants were given tenofovir disoproxil fumarate (300 mg) co-formulated with emtricitabine (200 mg) or lamivudine (150 mg). Follow-up visits were scheduled at week 4, week 12, and every 12 weeks thereafter for up to 144 weeks. We provided a supportive delivery system with options for visits at clinic, home, or community locations of the participants’ choice (eg, homes, near schools, trading centres, beaches). Visit procedures included evaluation of adverse events, self-assessed HIV risk (“are you currently at risk for HIV?”), HIV antibody testing, and PrEP refill, plus creatinine testing at week 12, week 24, and every 24 weeks thereafter. Participants who stopped PrEP were offered HIV testing and the opportunity to restart PrEP at each visit. Study drug and procedures were offered at no cost to the participants; no incentives were provided.

For adherence measurement at follow-up visits, we assessed self-reported adherence using 3-day recall,^15^ a feasible-to-collect, short-term measure. Given the limitations of self-reporting (eg, recall and social desirability biases, white-coat dosing) among participants reporting any adherence to PrEP (at least one dose of the past three), we collected small hair samples (around 50–100 strands) at each visit (week 4, week 12, and every 12 weeks thereafter) for adherence measurement via analysis of concentrations of tenofovir. We analysed tenofovir concentrations in hair among a random sample of participants who self-reported PrEP adherence and HIV risk at week 4, and in a random sample of participants who self-reported adherence and risk at week 24; sampling was stratified by age, sex, serodifferent partnership, and occupation (fishing, bar, or transport). 1 cm of hair closest to the scalp (reflecting the most recent 4 weeks of drug exposure) was analysed via liquid chromatography-tandem mass spectrometry using validated methods.^16^

### Statistical analysis

We calculated the following at the different steps of the PrEP cascade: (1) among all residents without HIV seen during population-level HIV testing, the proportion at elevated HIV risk (based on mutually exclusive categories, defined by serodifferent partnership, risk score without serodifferent partnership, or otherwise self-identified risk); (2) among individuals at elevated risk, the proportion with PrEP uptake, defined as initiation (receipt of pills) within 90 days of HIV testing during population-level testing; (3) among individuals who initiated PrEP within 90 days, programme engagement up to 72 weeks. Programme engagement was defined as follow-up visit attendance during periods defined on the basis of the date of PrEP enrolment: week 4 (−14/+28 days); week 12 (−27/+42 days); and weeks 24–72 (−41/+42 days). We censored at death or study withdrawal.

At each follow-up visit, we assessed the proportion of individuals who (1) received PrEP medication refills and (2) self-reported adherence to PrEP (at least one dose of the past three scheduled doses, assuming the participants who were not seen were non-adherent), both among all individuals who initiated PrEP and among individuals reporting current HIV risk. We also assessed adherence on the basis of tenofovir concentrations in hair from a sample of individuals to estimate the number of PrEP doses taken per week.^16,17^ Among individuals who stopped PrEP (no refill in visit period or refill ≥30 days late), we assessed the proportion of subsequent visits where HIV testing was done, and the proportion of participants who restarted PrEP.

We used mixed effects logistic regression with community as random effect and variances adjusted for clustering at the community level to identify factors associated with PrEP uptake (overall and stratified by sex) among participants at elevated HIV risk; week-24 self-reported adherence among individuals who initiated PrEP; and week-4 tenofovir concentrations in the hair of participants reporting risk using inverse weighting to account for sampling for measurement of tenofovir concentrations (appendix p 5) using Stata version 15. The SEARCH study is registered with ClinicalTrials.gov, NCT01864603.

### Role of the funding source

The funders of the study had no role in study design, data collection, data analysis, data interpretation, or writing of the report. The corresponding author had full access to all the data in the study and had final responsibility for the decision to submit for publication.

## Results

**Figure 1:**
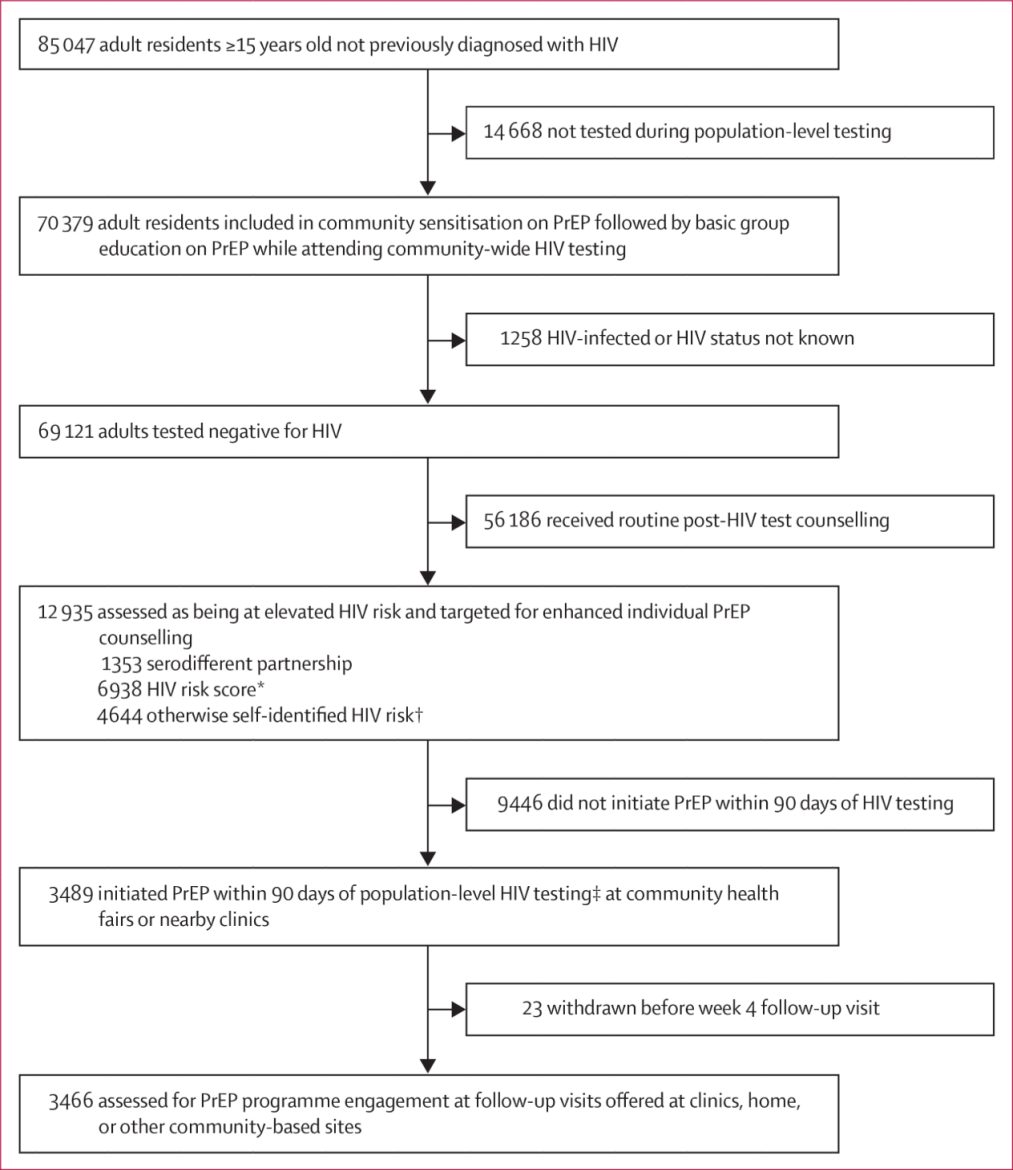
PrEP uptake after population-level HIV testing in 16 communities in rural Kenya and Uganda PrEP=pre-exposure prophylaxis. SEARCH=Sustainable East Africa Research in Community Health. *Empirical risk score developed on the basis of applying ensemble supervised machine learning methods to HIV seroconversion data from the first 2 years of the SEARCH test-and-treat trial, with a threshold selected to correctly classify 50% of seroconversions as at elevated risk across the three regions and minimise the number of individuals classified as at risk. Variables: age, sex, marital status, polygamy, education, occupation, alcohol, and circumcision. †Individuals neither in serodifferent partnerships nor identified by the risk score could self-identify as at risk of HIV acquisition. ‡82% initiated PrEP on the same day as seen during population-level HIV testing.

Between June 6, 2016, and June 23, 2017, 70 379 (83%) of 85 047 community residents 15 years or older and not previously diagnosed with HIV were tested during population-level HIV testing. Of the individuals who were tested, 69 121 residents tested negative for HIV, of whom 12 935 were assessed as having an elevated risk of HIV acquisition on the basis of serodifferent partnership (1353 [10%]), risk score (6938 [54%]), or otherwise self-identified risk (4644 [36%]) and were targeted for enhanced individual counselling on PrEP (figure 1). 820 (61%) of 1353 individuals in serodifferent partnerships and 2316 (32%) of all 7256 individuals identified by risk score also self-identified as being at risk (appendix p 2).

We measured PrEP uptake among the 12 935 people testing negative for HIV who were assessed to be at elevated HIV risk: 3489 (27%) initiated PrEP within 90 days of population-level testing; 2865 (82%) of whom started PrEP on the same day as testing. Among individuals who initiated PrEP, 1733 (50%) were men, 2175 (62%) were younger than 35 years, and 3395 (97%) were tested at health fairs rather than at home (table 1). Compared with women who initiated PrEP, men were more likely to be unmarried, use alcohol, and work in fishing, bar, or transport occupations (appendix p 3). Among individuals who were assessed to be at elevated HIV risk, 446 (19%) of 2376 adolescent girls and young women aged 15–24 years, 1226 (17%) of the 7256 individuals identified by risk score, and 3374 (45%) of 7581 individuals with self-identified HIV risk initiated PrEP (appendix p 4). Of 1353 serodifferent partners, 603 (45%) initiated PrEP (235 [39%] of 603 men; 368 [49%] of 750 women); 192 (26%) of 750 who did not initially start PrEP started after 90 days. Of 1014 HIV-negative residents linked to a serodifferent spouse, 729 (72%) were in a partnership for at least 3 years with a spouse living with HIV. Among 811 spouses living with HIV with HIV-RNA measured during population-level testing, 246 (82%) of 299 spouses of individuals who initiated PrEP had viral suppression (≤500 copies per mL) compared with 410 (80%) of 512 spouses of participants who had not initiated PrEP. Among individuals who initiated PrEP and were linked to a serodifferent spouse, 238 (68%) of 351 (165 [71%] of 231 women; 73 [61%] of 120 men) reported a serodifferent partnership at PrEP enrolment, suggesting that disclosure of HIV status might have occurred.

We analysed predictors of PrEP uptake among 12 850 individuals assessed to have elevated HIV risk with complete covariate data (table 2). The adjusted odds of PrEP uptake were significantly higher among individuals in serodifferent partnerships, polygamous marriages, or who were divorced, separated, or widowed, and were significantly lower among participants aged 15–24 years and 25–34 years, and among mobile individuals (mobility was defined as migration out of the community for at least 1 month, or moving residence within the past 12 months). In analyses stratified by sex, non-single marital status was associated with PrEP uptake for both men and women (table 3). Among women, the adjusted odds of PrEP uptake were significantly lower for individuals with fishing, bar, or transport work, alcohol consumption, or mobility, and were higher among serodifferent partners.

**Figure 2:**
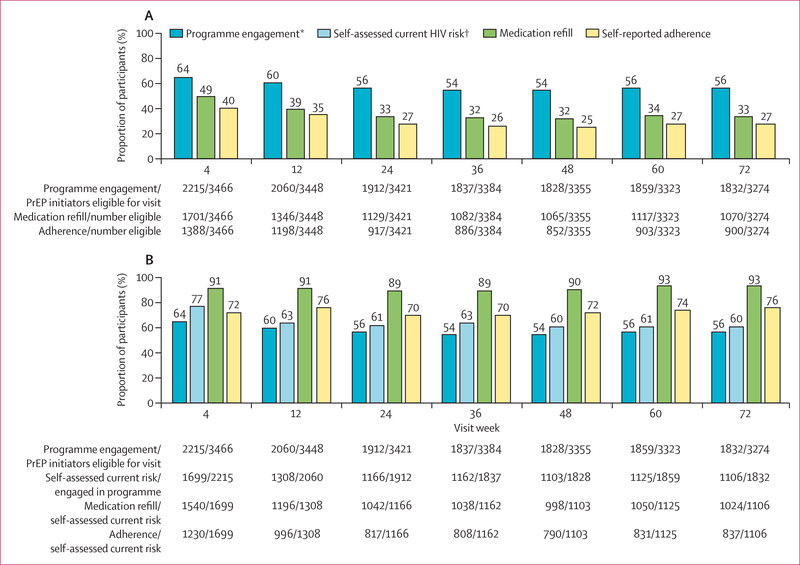
PrEP programme engagement, refill, and adherence overall and by self-assessed current HIV risk up to week 72 (A) PrEP programme engagement, refill, and self-reported adherence among PrEP initiators. (B) Refill and self-reported adherence among PrEP participants reporting self-assessed current HIV risk. PrEP=pre-exposure prophylaxis. *Programme engagement is defined as attendence at a PrEP follow-up visit during scheduled visit weeks; eligibility for visit excludes participants who were withdrawn or died before the visit. †Self assessed current HIV risk was evaluated at each follow-up visit among individuals engaged in the PrEP programme.

We measured PrEP programme engagement, refills, and adherence up to June 20, 2019, among 3466 individuals (excludes participants who were withdrawn or died before the first visit) who had initiated PrEP; 2693 (78%) of 3466 attended at least one follow-up visit. At week 4, 2215 (64%) individuals who initiated PrEP were engaged in the PrEP programme; 1701 (49%) received PrEP refills; and 1388 (40%) self-reported adherence to PrEP (at least one dose of the past three), of whom 1268 (91%) reported that all of the past three doses were taken (figure 2A). Programme engagement, refills, and self-reported adherence declined until week 24, then stabilised up to week 72. At week 72, 1832 (56%) of 3274 were engaged, 1070 (33%) received a refill, and 900 (27%) self-reported adherence. Over time, more follow-up visits occurred at home and community sites (vs at facilities): 1311 (59%) of 2215 at week 4, 1443 (76%) of 1912 at week 24, and 1459 (80%) of 1832 at week 72. In multivariable analyses of factors associated with self-reported PrEP adherence at week 24, self-assessed current HIV risk was associated with the greatest odds of adherence; serodifferent partnership, or being divorced, separated, or widowed were positively associated with adherence; and being aged 15–24 years was negatively associated with adherence (table 4).

Among the subset of individuals who initiated PrEP and reported current HIV risk during follow-up, a high proportion received PrEP refills and self-reported adherence. At weeks 4–72, 89–93% received refills and 70–76% self-reported adherence (figure 2B). Self-assessed risk also changed over time: 1462 (54%) of 2693 participants seen in follow-up reported no risk at at least one visit. Of 1699 participants reporting current HIV risk at week 4, 330 (19%) reported no risk at week 24; conversely, 96 (20%) of 471 participants who reported no risk at week 4 reported risk at week 24.

**Figure 3:**
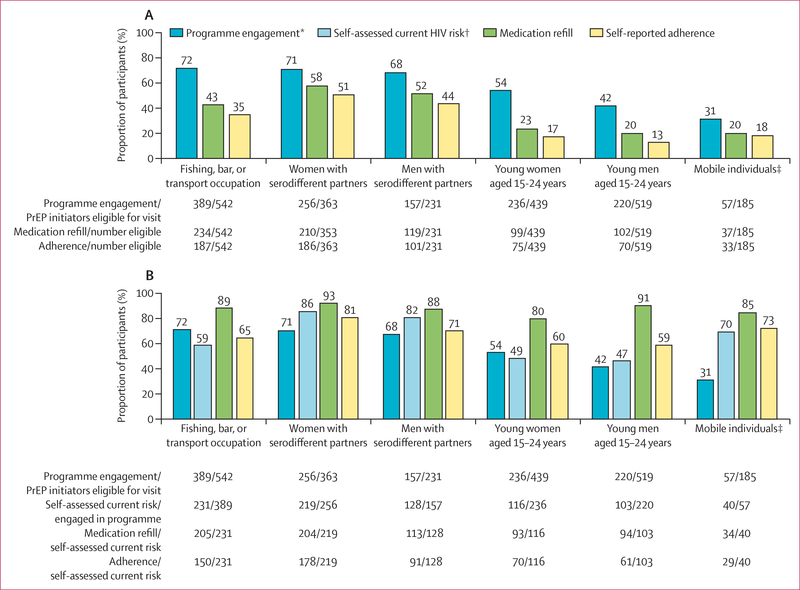
PrEP programme engagement, refill, and adherence at week 24 among demographic subgroups of participants who initiated PrEP (A) PrEP programme engagement, refill, and self-reported adherence among risk groups of participants who initiated PrEP. (B) Refill and self-reported adherence among PrEP participants reporting self-assessed current HIV risk. PrEP=pre-exposure prophylaxis. *Programme engagement is defined as attendence at a PrEP follow-up visit during scheduled visit weeks; eligibility for visit excludes participants who were withdrawn or died before the visit. †Self-assessed current HIV risk was evaluated at each follow-up visit among individuals engaged in the PrEP programme. ‡Mobile individuals could be in any age group.

Among subpopulations considered to be at higher risk of HIV, we examined cascade outcomes at week 24. adults reported current HIV risk versus 219 (86%) of 256 women with serodifferent partners (figure 3B).

**Figure 4:**
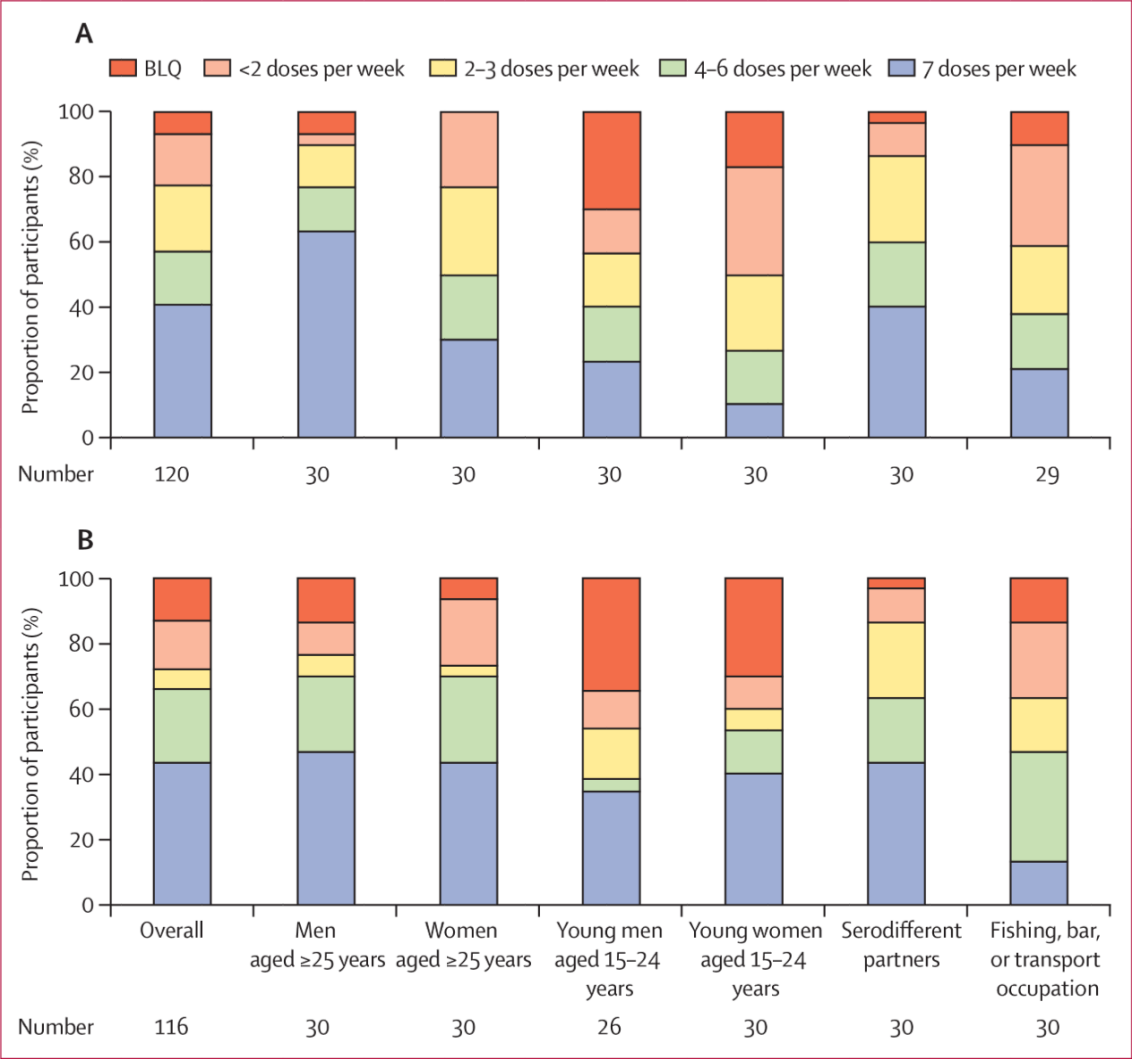
Adherence to PrEP estimated from the concentration of tenofovir in hair samples Adherence at (A) week 4 (n=166) and (B) week 24 (n=152) among subgroups of sampled participants reporting self-assessed current HIV risk and any PrEP adherence (at least one dose taken of the past three). BLQ=below the limit of quantification.

We analysed concentrations oftenofovir in hair samples from a sample of individuals who initiated PrEP and who reported current risk and any adherence. After accounting for sampling weights for 120 participants with tenofovir concentrations measured at week 4, 57% (95% CI 47– 67) had tenofovir concentrations consistent with taking at least four PrEP doses per week; 40% (31–51) had tenofovir concentrations consistent with taking seven PrEP doses per week (figure 4). Adherence of at least four doses per week was highest among men aged 25 years and older (77%, 58–90) and lowest among young women aged 15–24 years (27%, 12–46). Participants sampled at week 24 had concentrations of tenofovir in hair samples consistent with similar or higher numbers of doses taken per week compared with those sampled at week 4, both overall and in most subgroups, including young women. After accounting for sampling weights for 116 participants with concentrations of tenofovir in hair samples measured at week 24, 66% (55–76) had tenofovir concentrations consistent with taking at least four doses per week, and 44% (33–55) had tenofovir concentrations consistent with taking seven doses per week. However, one-third (34%, 24–45) of individuals who initiated PrEP had drug concentrations that suggest poor adherence (fewer than four doses per week). Among individuals who initiated PrEP and reported risk at week 4, female sex and being aged 15–24 years were associated with lower odds of having concentrations of tenofovir consistent with seven doses per week; serodifferent partnership was associated with the greatest odds (appendix p 5). When analysed as a continuous outcome, higher tenofovir concentrations were associated with serodifferent partnership, whereas younger age was associated with lower tenofovir concentrations (appendix p 6).

Many individuals who initiated PrEP stopped the drug and later restarted. Among 2693 participants seen in follow-up, 2205 (82%) stopped PrEP at least once; 1096 (50%) restarted by week 72. Among participants who restarted, 201 (18%) stopped and restarted more than once and the median time off PrEP was 68 days (IQR 37–188). HIV testing was done at 4274 (83%) of 5140 visits where participants had stopped PrEP before the visit or did not receive a refill.

29 of 3489 participants who initiated PrEP had serious adverse events, including seven deaths (table 5, appendix p 7). Five adverse events (all grade 3) were assessed as being possibly related to the study drug. One grade 3 creatinine elevation occurred in a 71-year-old man who was treated in hospital for urinary retention and hydronephrosis. Creatinine returned to baseline following relief of urinary obstruction and cessation of the study drug.

## Discussion

During HIV testing of more than 70000 individuals across three regions of rural Kenya and Uganda, we evaluated a population-based approach to engage people in PrEP that included community sensitisation, group-based education, individual counselling, real-time risk-score assessment, and universal PrEP access with flexibility in refill location. More than 12 000 individuals were assessed to be at elevated risk of HIV, of whom nearly 3500 initiated PrEP within 90 days of population-level HIV testing. The greatest gap in the PrEP cascade was initial uptake, with only 27% starting PrEP; adolescents, young adults, and mobile individuals had the lowest uptake. Programme engagement up to 72 weeks was high (56%) among individuals who initiated PrEP, with 78% of these individuals having at least one follow-up visit and most engaging in visits for HIV testing. Receipt of PrEP refills and self-reported adherence were much higher among participants who perceived themselves to be at ongoing risk at follow-up visits. However, one-third of participants who self-reported adherence had drug concentrations that indicated low adherence.

We posited that universal access to PrEP and inclusive risk assessment could provide an opportunity for PrEP initiation for individuals who might not otherwise engage with health systems. Moreover, given concerns that targeting PrEP towards specific risk groups could stigmatise use,^18^ offering PrEP more broadly could foster uptake. Although we have no comparator for other population-level approaches, only one-quarter of individuals assessed as at elevated HIV risk initiated PrEP within 90 days. Our study rapidly introduced PrEP in communities before national roll-out in Kenya and Uganda, and the novelty of PrEP might have mitigated against higher uptake. We have previously reported on barriers to PrEP uptake in study communities, including myths and misconceptions about this new drug, unsupportive partners, daily pilltaking, fear of side-effects, and anticipated stigma,^19^ similar to previous studies.^20^ We have also found that distance to clinic is a barrier to PrEP uptake and engagement.^21^ Moreover, because an individual’s HIV risk is potentially dynamic,^22^ some individuals assessed as being at elevated HIV risk at screening might not have had current sexual risk factors or partners at the time of PrEP screening. Finally, although we offered universal PrEP access, our approach focused on risk assessment. Wellness framing of PrEP (as fostering protection and sexual health and wellbeing) might be more successful than risk framing^18^ and could be evaluated in future studies.

Our study provides insights into PrEP uptake in several subpopulations, including adolescents and young adults, men, and serodifferent partners, using a population-level denominator. In our study communities^9^ and across sub-Saharan Africa, adolescent girls and young women have among the highest HIV risk, yet they were less likel than women aged 25 years or older to initiate PrEP. A barrier for adolescents and young adults in our study was that these individuals were more likely to be mobile than older people. In our qualitative work, adolescents and young adults who initiated PrEP were motivated by high perceived HIV risk and beliefs that PrEP use supported pursuing life goals, but some wanted “proof”^19^ of PrEP efficacy. Further mixed-methods investigation of these barriers is warranted.

Men have largely not been prioritised for PrEP in sub-Saharan Africa, except for men in serodifferent couples and men who have sex with men.^23^ Yet, one-third of new HIV diagnoses in our study region are among men.^9^ We found substantial PrEP uptake among men, who comprised half of the individuals who initiated PrEP. In our qualitative work, we have found high levels of interest in PrEP among men, particularly among those in concurrent partnerships, and found that men typically face fewer barriers to PrEP adoption (eg, partner permission) than women.^19^ Our approach to PrEP service delivery, with out-of-facility options for PrEP initiation and follow-up, might have fostered uptake among men, for whom clinic attendance might pose a barrier to HIV testing and prevention services.^24^ Men are a crucial link in transmission networks,^25^ and expanding their prevention options could accelerate incidence reductions in generalised epidemics.

PrEP uptake was higher among serodifferent partners than among other groups in this study, yet fewer than half of individuals in serodifferent partnerships started PrEP within 90 days of population-level HIV testing. Our study population contrasts with previous studies that have reported high levels of PrEP initiation among mutually disclosed serodifferent couples presenting to clinics before ART eligibility or initiation.^2,8^ We approached serodifferent partners at a population level, who might not have tested together or disclosed their HIV status. We did intensive outreach to serodifferent couples during population-level testing and at clinics, where couples’ counselling was routinely offered, but some partners were not residing in or in care within the study communities. We also found that, overall, 80% of spouses with HIV were virally suppressed and 70% were in serodifferent partnerships for at least 3 years. These factors could have reduced the perceived need for couples to initiate PrEP. Finally, we observed sex differences in PrEP uptake among individuals without HIV in serodifferent partnerships: 49% of women and 39% of men initiated PrEP. This difference could have been related to the interest of women in discreet, female-controlled HIV prevention, or to barriers to disclosure among women with HIV. As ART and viral-load monitoring are scaled up, counselling messages for serodifferent partners^26^ should be tailored on the basis of the durability of viral suppression and barriers to ART adherence for the partner who has HIV, and whether the partner without HIV infection has concurrent partners who might be viraemic or of unknown HIV status.

Among participants in our study who initiated PrEP, engagement at follow-up visits was among the highest reported to date in Kenya and Uganda in studies not solely enrolling serodifferent couples.^6,23,27,28^ Three-quarters of ndividuals who initiated PrEP were seen at least once during follow-up, and more than half remained engaged in the PrEP programme up to 72 weeks. Medication refill (≥89%) and self-reported adherence (≥70%) were high among participants reporting HIV risk during follow-up, but more than half of participants who initiated PrEP reported no current HIV risk at least once, and many had breaks in PrEP use. Barriers to PrEP engagement and adherence were similar to barriers to uptake, but reasons for stopping also included changes in partnerships and risk perception.^19^ Among individuals who stopped PrEP, ongoing programme engagement provided opportunities for repeat HIV testing and PrEP access, and half of the participants who had stopped PrEP later restarted the drug. As prevention options expand to other modalities, such as long-acting PrEP, follow-up visits could serve as platforms for frequent HIV testing and delivery of a variety of HIV prevention options.

Although at least 70% of individuals reporting current HIV risk at each follow-up visit self-reported adherence to PrEP, concentrations of tenofovir in hair samples showed poor adherence (fewer than four doses per week) in one-third of those individuals. Of particular concern is that women and participants younger than 25 years had lower odds of having tenofovir concentrations consistent with daily dosing (which might be required for protection from vaginal exposure to HIV)^29^ at week 4. We did find higher adherence among young women sampled at week 24, but only slightly more than half had tenofovir concentrations consistent with taking at least four doses per week. Given that many participants had breaks in PrEP use, concentrations of tenofovir in hair could have underestimated adherence among individuals restarting PrEP in the preceding 30 days. Although data on cumulative tenofovir concentrations are limited in eastern Africa, studies among young women in southern Africa^30,31^ found that half or fewer had tenofovir-diphosphate concentrations in dried blood spots consistent with at least four doses per week, and a minority of participants had concentrations consistent with daily PrEP use.

To our knowledge, this study is the first in sub-Saharan Africa to implement a machine learning-based risk score to identify individuals for PrEP initiation. We found that real-time entry of demographic data and score assessment at the point-of-contact during community-based HIV testing was feasible. In generalised epidemic settings, machine learning-based scores have shown the potential to improve the efficiency of identifying individuals for enhanced prevention compared with model-based scores or targeting traditional risk groups.^32^ Further research is needed on optimal approaches for implementing risk scores (and results counselling), and comparing risk scores to risk self-assessment and guidelines-based eligibility. Importantly, our risk score was not used to exclude individuals from PrEP eligibility but to foster conversations about PrEP. Individuals not identified by risk score who self-identified as being at risk (regardless of serodiscordance or risk score) could access PrEP, in line with WHO guidance to offer PrEP to those requesting it.^33^

A major strength of this study is the use of population-based HIV testing and risk assessment, which enabled us to establish a population-level denominator for PrEP cascade analyses. Our approach achieved high testing coverage,^11^ allowing individuals to rapidly learn their status and seek ART or PrEP. As ART coverage and viral suppression increase, achieving epidemic control will require intensified HIV prevention for individuals who remain at risk. Questions remain, however, about the impact and cost-effectiveness of population-level PrEP roll-out, and the levels of uptake and adherence needed to reduce HIV incidence.

This study has several limitations. During the population-level testing window, 17% of community members did not have HIV testing or a risk assessment. The risk score did not include questions about sexual behaviour, sex work, or same-sex partnerships, although we also allowed for risk self-identification. Future research should evaluate whether sexual behaviour data improve risk score performance (and optimal methods to facilitate disclosure and discussions on sexual health). Additionally, written consent was required to initiate PrEP, which might limit generalisability to settings now offering PrEP within routine care.

Rapid introduction of universal PrEP access during population-level HIV testing resulted in nearly 3500 PrEP starts within 90 days in rural Kenya and Uganda. We found that a substantial proportion of the population want PrEP and can take it, including many who would not have otherwise had access to PrEP without inclusive eligibility. As PrEP roll-out accelerates, our study provides insights into gaps in the PrEP cascade that can be addressed to optimise the impact of this powerful prevention tool. Combination approaches to HIV prevention (including oral PrEP and, ultimately, on-demand and long-acting methods) that are inclusive and offer flexible delivery, hold promise to ultimately reduce HIV incidence in generalised epidemic settings.

## Supplementary Material

Appendix

## Figures and Tables

**Table 1:** Characteristics of adult residents who tested negative for HIV infection, individuals assessed to be at elevated HIV risk, and individuals who initiated PrEP within 90 days of HIV testing in 16 communities in rural Kenya and Uganda

	Residents who tested negative for HIV infection (n=69 121)	Residents without HIV infection at elevated HIV risk (n=12 935)	Residents without HIV infection with PrEP uptake within 90 days of HIV testing (n=3489)
**Sex**			
Male	31 276 (45%)	6476 (50%)	1733 (50%)
Female	37 845 (55%)	6459 (50%)	1756 (50%)
**Age, years**			
15–24	25 562 (37%)	4800 (37%)	978 (28%)
25–34	14 739 (21%)	4712 (36%)	1197 (34%)
35–44	10 236 (15%)	1927 (15%)	752 (22%)
45–54	7177 (10%)	991 (8%)	393 (11%)
≥55	11 407 (17%)	505 (4%)	169 (5%)
**Educational attainment** *			
Less than primary level	8640 (13%)	716 (6%)	224 (6%)
Primary school level	42 154 (61%)	8559 (66%)	2371 (68%)
Any secondary school level and higher	18 255 (26%)	3637 (28%)	890 (26%)
**Occupation** †			
Farmer	35 634 (52%)	5578 (43%)	1795 (51%)
Student	13 932 (20%)	853 (7%)	170 (5%)
Fishing, bar, or transportation	3421 (5%)	2349 (18%)	548 (16%)
Other informal sector	8721 (13%)	2567 (20%)	643 (18%)
Other formal sector	2972 (4%)	713 (6%)	149 (4%)
Unemployed or disabled	4081 (6%)	777 (6%)	164 (5%)
Other or unknown	321 (0·5%)	87 (1%)	16 (0·5%)
**Marital status** ‡			
Not married	20 624 (30%)	3669 (28%)	721 (21%)
Married (monogamous)	33 595 (49%)	6428 (50%)	1788 (51%)
Married (polygamous)	7396 (11%)	1993 (15%)	692 (20%)
Divorced, separated, or widowed	7466 (11%)	833 (6%)	285 (8%)
**Circumcised** §			
By a health-care provider	7061 (23%)	1809 (28%)	513 (30%)
By a traditional practitioner	5748 (18%)	1121 (17%)	317 (18%)
Uncircumcised	18 405 (59%)	3489 (54%)	898 (52%)
**Alcohol use** ¶			
None	59 979 (87%)	10 600 (82%)	2823 (81%)
1–7 days per month	3743 (5%)	1079 (8%)	257 (7%)
>7 days per month	5366 (8%)	1241 (10%)	408 (12%)
**Mobility** ∥			
Yes	4464 (6%)	1131 (9%)	192 (6%)
**HIV testing site**			
Community health fair	57 107 (83%)	10 680 (83%)	3395 (97%)
Home-based testing	12 014 (17%)	2255 (17%)	94 (3%)
**Region**			
Western Kenya	19 321 (28%)	6418 (50%)	1542 (44%)
Eastern Uganda	25 159 (36%)	2871 (22%)	930 (27%)
Western Uganda	24 641 (36%)	3646 (28%)	1017 (29%)

Data are number (%). PrEP=pre-exposure prophylaxis.

* Missing data for 72 residents (0·10%).

† Missing data for 39 residents (0·06%); other formal sector occupations are teaching, government, military, health care, and factory; other informal sector occupations are shopkeeper, market vendor, hotel worker, homemaker, household worker, miner, and construction.

‡ Missing data for 40 residents (0·06%).

§ Assessed among 31 276 men; missing data for 62 residents (0·20%).

¶ Missing data for 33 residents (0·05%).

∥ Missing data for 547 residents (0·8%); mobility is migration out of the community for at least 1 month or moved residence within the past 12 months; among individuals at elevated risk, mobility was more prevalent among participants aged 15–24 years (13%) versus participants aged 35–44 years (5%).

**Table 2:** Factors associated with PrEP uptake among adult residents assessed to be at elevated HIV risk in 16 communities in rural Kenya and Uganda

	Odds ratio (95% CI)	p value	Adjusted odds ratio (95% CI)	p value
**Sex**				
Male	1	··	1	··
Female	1·04 (0·86–1·24)	0·70	0·88 (0·75–1·05)	0·16
**Age, years**				
15–24	0·41 (0·34–0·49)	<0·0001	0·55 (0·45–0·68)	<0·0001
25–34	0·53 (0·45–0·63)	<0·0001	0·61 (0·52–0·72)	<0·0001
35–44	1	··	1	··
≥45	0·94 (0·80–1·09)	0·40	0·86 (0·73–1·00)	0·052
**Marital status**				
Not married	1	··	1	··
Married (monogamous)	1·65 (1·36–1·99)	<0·0001	1·19 (0·96–1·48)	0·11
Married (polygamous)	2·31 (2·02–2·63)	<0·0001	1·54 (1·29–1·84)	<0·0001
Divorced, separated, or widowed	2·05 (1·68–2·50)	<0·0001	1·59 (1·30–1·95)	<0·0001
**Serodifferent partnership**				
No or unknown	1	··	1	··
Yes	2·57 (1·90–3·47)	<0·0001	2·02 (1·44–2·84)	0·0005
**Occupation** *				
Student or other formal sector occupation	1	··	1	··
Fishing, bar, or transport	1·25 (0·94–1·66)	0·12	0·85 (0·60–1·20)	0·34
Farming or other informal sector occupation	1·66 (1·37–2·00)	<0·0001	1·16 (0·91–1·48)	0·23
Unemployed, disabled, or other	1·13 (0·91–1·41)	0·27	0·98 (0·77–1·26)	0·89
**Educational attainment**				
Less than primary level	1	··	1	··
Primary school level	0·87 (0·69–1·11)	0·27	1·07 (0·87–1·31)	0·54
Any secondary school level or higher	0·68 (0·52–0·90)	0·0078	1·04 (0·80–1·35)	0·78
**Alcohol use**				
None	1	··	1	··
At least 1 day per month	1·04 (0·89–1·23)	0·62	0·96 (0·81–1·13)	0·59
**Mobility in the past 12 months** †				
No	1	··	1	··
Yes	0·54 (0·37–0·79)	0·0015	0·61 (0·41–0·91)	0·016

Mixed effects logistic regression with community as random effect and variances adjusted for clustering at community level. Of 12 935 residents at elevated HIV risk, 12 850 were included in the analysis; 85 individuals (0·7%) were excluded because of missing data on covariates. PrEP=pre-exposure prophylaxis.

* Other formal sector occupations are teaching, government, military, health care, and factory; other informal sector occupations are shopkeeper, market vendor, hotel worker, homemaker, household worker, miner, and construction.

† Mobility is migration out of the community for at least 1 month or moved residence within the past 12 months.

**Table 3:** Factors associated with PrEP uptake stratified by sex among men and women assessed to be at elevated HIV risk in 16 communities in rural Kenya and Uganda

	Men (n=6426)		Women (n=6424)	
	Adjusted odds ratio (95% CI)	p value	Adjusted odds ratio (95% CI)	p value
**Age, years**				
15–24	0·74 (0·56–0·97)	0·027	0·43 (0·33–0·56)	<0·0001
25–34	0·72 (0·60–0·86)	0·0003	0·52 (0·43–0·63)	<0·0001
35–44	1	··	1	··
≥45	1·03 (0·84–1·27)	0·75	0·79 (0·61–1·01)	0·065
**Marital status**				
Not married	1	··	1	··
Married (monogamous)	1·13 (0·87–1·47)	0·35	1·60 (1·19–2·13)	0·0016
Married (polygamous)	1·51 (1·15–1·99)	0·0030	1·86 (1·45–2·40)	<0·0001
Divorced, separated, or widowed	2·15 (1·56–2·96)	<0·0001	1·70 (1·30–2·22)	0·0001
**Serodifferent partnership**				
No or unknown	1	··	1	··
Yes	1·54 (0·96–2·45)	0·072	2·41 (1·80–3·22)	<0·0001
**Occupation** *				
Student or other formal sector occupation	1	··	1	··
Fishing, bar, or transport	0·85 (0·56–1·28)	0·44	0·68 (0·52–0·89)	0·0048
Farming or other informal sector occupation	1·17 (0·89–1·55)	0·27	1·02 (0·82–1·29)	0·82
Unemployed, disabled, or other	0·86 (0·60–1·24)	0·42	1·02 (0·78–1·32)	0·90
**Educational attainment**				
Less than primary level	1	··	1	··
Primary school level	0·99 (0·78–1·27)	0·95	1·21 (0·94–1·55)	0·14
Any secondary school level or higher	1·11 (0·81–1·50)	0·53	0·97 (0·72–1·29)	0·81
**Alcohol use**				
None	1	··	1	··
At least 1 day per month	1·09 (0·94–1·25)	0·24	0·74 (0·55–0·99)	0·040
**Mobility in the past 12 months** †				
No	1	··	1	··
Yes	0·67 (0·45–1·01)	0·059	0·54 (0·35–0·83)	0·0049

Mixed effects logistic regression with community as random effect and variances adjusted for clustering at community level. Of 12 935 residents (6476 men and 6459 women) at elevated HIV risk, 12 850 (6426 men and 6424 women) were included in the analysis; 85 (0·7%) residents were excluded because of missing data on covariates. PrEP=pre-exposure prophylaxis.

* Other formal sector occupations are teaching, government, military, health care, and factory; other informal sector occupations are shopkeeper, market vendor, hotel worker, homemaker, household worker, miner, and construction.

† Mobility is migration out of the community for at least 1 month or moved residence within the past 12 months.

**Table 4:** Factors associated with self-reported adherence to PrEP among individuals who initiated PrEP and were seen at a week 24 follow-up visit in 16 communities in rural Kenya and Uganda

	Odds ratio (95% CI)	p value	Adjusted odds ratio (95% CI)	p value
**Sex**				
Male	1	··	1	··
Female	1·07 (0·88–1·30)	0·51	0·83 (0·63–1·09)	0·17
**Age, years**				
15–24	0·37 (0·28–0·50)	<0·0001	0·59 (0·40–0·86)	0·0067
25–34	0·67 (0·52–0·87)	0·0030	0·86 (0·63–1·17)	0·33
35–44	1	··	1	··
≥45	0·87 (0·64–1·18)	0·37	0·98 (0·68–1·41)	0·90
**Marital status**				
Not married	1	··	1	··
Married (monogamous)	1·69 (1·26–2·27)	0·0005	1·23 (0·81–1·88)	0·33
Married (polygamous)	2·46 (1·76–3·45)	<0·0001	1·41 (0·87–2·28)	0·17
Divorced, separated, or widowed	2·91 (1·80–4·72)	<0·0001	2·10 (1·12–3·95)	0·021
**Serodifferent partnership**				
No or unknown	1	··	1	··
Yes	2·76 (2·14–3·55)	<0·0001	1·64 (1·22–2·19)	0·0009
**Occupation** *				
Student or other formal sector occupation	1	··	1	··
Fishing, bar, or transport	1·69 (1·10–2·57)	0·015	0·83 (0·47–1·47)	0·52
Farming or other informal sector occupation	1·57 (1·09–2·28)	0·016	0·88 (0·53–1·49)	0·64
Unemployed, disabled, or other	2·01 (1·21–3·34)	0·0071	1·52 (0·79–2·93)	0·21
**Educational attainment**				
Less than primary level	1	··	1	··
Primary school level	0·81 (0·52–1·27)	0·36	0·87 (0·51–1·45)	0·59
Any secondary school level or higher	0·60 (0·37–0·97)	0·037	0·65 (0·36–1·16)	0·14
**Alcohol use**				
None	1	··	1	··
At least 1 day per month	1·05 (0·77–1·43)	0·77	0·81 (0·55–1·18)	0·27
**Mobility in past 12 months** †				
No	1	··	1	··
Yes	1·12 (0·61–2·04)	0·71	1·12 (0·56–2·26)	0·74
**Self-assessed current HIV risk**				
No	1	··	1	··
Yes	13·46 (10·30–17·59)	<0·0001	12·36 (9·39–16·28)	<0·0001

Mixed effects logistic regression with community as random effect and variances adjusted for clustering at community level. Of 3421 individuals who initiated PrEP who were alive and not withdrawn at week 24, 1912 attended a week 24 visit, of whom 1863 are included in this analysis. 49 participants (2·6%) had missing data on covariates and are excluded from this analysis. PrEP=pre-exposure prophylaxis.

* Other formal sector occupations are teaching, government, military, health care, and factory; other informal sector occupations are shopkeeper, market vendor, hotel worker, homemaker, household worker, miner, and construction.

† Mobility is migration out of the community for at least 1 month or moved residence within the past 12 months.

**Table 5:** Adverse events among 3489 individuals who initiated pre-exposure prophylaxis

	Total (n=3489)
Any grade 3 or 4 adverse event	28 (0·8%)
Grade 3 creatinine elevation	1 (0·03%)
Grade 4 creatinine elevation	0
Grade 3 adverse event possibly related to the study drug	5 (0·1%)
Grade 4 adverse event possibly related to the study drug	0
Any serious adverse event	29 (0·8%)
Death	7 (0·2%)

Grade 3 or 4 adverse events, serious adverse events, and causes of death are listed in the appendix (p 7). Adverse events possibly related to the study drug were creatinine elevation (n=1), dizziness (n=2), fatigue (n=1), and headache (n=1). All other grade 3 or 4 adverse events were judged to be unlikely (n=2) or not related to the study drug. One serious adverse event was judged to be possibly related to the study drug: a 71-year-old man was treated in hospital for urinary retention and found to have bilateral hydronephrosis and grade 3 creatinine elevation. Creatinine returned to baseline following relief of urinary obstruction and cessation of the study drug.

## Research in context

### Evidence before this study

Oral pre-exposure prophylaxis (PrEP) is highly effective and roll-out is expanding worldwide, including across sub-Saharan Africa. In generalised epidemic settings, strategies are needed to identify and engage individuals who might benefit from HIV prevention services, including PrEP. We searched PubMed using the terms “HIV” and (“pre-exposure prophylaxis”, or “preexposure prophylaxis”, or “PrEP”) and (“Africa”, or “Kenya”, or “Uganda”) between Jan 1, 2010, and Aug 7, 2019. We did not use any language restrictions. We also examined abstracts from the Conference on Retroviruses and Opportunistic Infections (2015–19), the International AIDS Conference/IAS Conference on HIV Science (2015–19), and HIV Research for Prevention (2016 and 2018). We excluded studies on cost-effectiveness, modelling, or PrEP acceptability without uptake data.

We reviewed national guidelines on PrEP and programme outcomes, where reported, from Kenya, Uganda, and South Africa from 2016 to 2019. Most research studies reported on PrEP uptake among specific populations at risk for HIV, such as serodifferent couples, young women, female sex workers, and men who have sex with men. These studies resulted in a wide range of estimates of PrEP uptake from individuals attending health facilities or after community-based recruitment of specific groups. National guidelines now allow for PrEP eligibility beyond members of key populations, but programmes have often reported PrEP initiation and follow-up visits within specific subpopulations at risk of HIV. No previous studies evaluated PrEP uptake or cascade outcomes at a population level in sub-Saharan Africa.

### Added value of this study

To our knowledge, this study is the first examination of a population-based strategy of universal PrEP access in sub-Saharan Africa in the context of community-wide HIV testing. Our testing approach enabled us to estimate the proportion of the entire population at elevated risk for HIV and thereby establish a denominator for calculations of PrEP uptake (rather than estimating uptake after clinic-based recruitment). A novel feature of our approach was the use of three methods to inclusively evaluate HIV risk and provide enhanced counselling on PrEP: serodifferent partnerships, self-identified risk, and real-time risk assessment using a machine learning-based score. We noted a particular gap in the PrEP cascade in terms of uptake: only one-quarter of the individuals assessed as being at elevated risk initiated PrEP within 90 days, with even lower uptake among young adults and mobile individuals. We also found lower uptake among serodifferent partners identified at a population level compared with previous studies of clinic-recruited, mutually disclosed couples. PrEP was delivered using a flexible model with options for out-of-facility PrEP visits: in this context, three-quarters of individuals who initiated PrEP attended at least one follow-up visit and more than half remained engaged in the PrEP programme at 72 weeks. However, a second notable gap in the cascade was in PrEP adherence. One-third of participants reporting HIV risk and adherence during follow-up had concentrations of tenofovir in the hair that were consistent with poor adherence (fewer than four PrEP doses per week), with even lower levels of adherence at week 4 among women and young adults.

### Implications of all the available evidence

Comprehensive HIV testing and universal access to PrEP for all individuals without HIV who are at elevated risk is one approach to offering PrEP to people who might not otherwise engage in health systems and to assessing PrEP engagement at a population level. As PrEP roll-out expands in generalised epidemic settings, our study provides insights into real-world implementation gaps in the PrEP cascade and suggests that further interventions are needed to maximise uptake and adherence, particularly among young adults and mobile populations. Novel strategies are needed to foster sustained engagement and adherence to oral PrEP and future long-acting formulations.
