# Supplementary material for: Uptake, engagement, and adherence to pre-exposure prophylaxis offered after population HIV testing in rural Kenya and Uganda: 72-week interim analysis of observational data from the SEARCH study
Source: Lancet HIV. Author manuscript; Available in PMC 2020 May 13. (PMC7208546; doi:10.1016/S2352-3018(19)30433-3)
Supplement: Appendix [file NIHMS1584758-supplement-Appendix.pdf]

THE LANCET
HIV

Supplementary appendix

This appendix formed part of the original submission and has been peer reviewed. We post it as supplied by the authors.

Supplement to: Koss CA, Charlebois ED, Ayieko J, et al. Uptake, engagement, and adherence to pre-exposure prophylaxis offered after population HIV testing in rural Kenya and Uganda: 72-week interim analysis of observational data from the SEARCH study. *Lancet HIV* 2020; published online Feb 19. https://doi.org/10.1016/S23523018(19)30433-3.

APPENDIX

Development of Machine Learning-based Risk Score

The empiric risk-score, used as one component of our inclusive approach to HIV risk assessment, has been previously described.[1,2] Briefly, the risk-score was developed using repeat HIV testing data collected from community members ≥15 years of age during annual population-level HIV testing (health fairs and home-based testing) in intervention communities during the first two years of the SEARCH test-and-treat trial.[3] We used as risk predictors region, age, sex, marital status, polygamy, education, occupation, alcohol, and circumcision. Data on sexual behavior, sex of partners, sexually transmitted infections, and self-assessed risk of HIV acquisition were not previously collected and thus not available for risk-score development.

The risk-score was developed by first classifying all persons in known serodifferent partnerships as at elevated risk and then applying machine-learning to build a risk classifier for HIV seroconversion among the remaining population.[2] Specifically, we used Super Learning, an ensemble method that constructs weighted combinations of multiple prediction algorithms based on internal sample splitting (cross validation).[4] Using the methods described in detail in Zheng et al.,[2] we extended this methodology to construct a risk classifier (consisting of a continuous risk prediction and a cutoff above which persons were classified at elevated risk) that was designed to minimize the rate of positive predictions under a constraint on sensitivity.[2] Across all regions combined, we selected a risk-score cutoff such that on independent data (evaluated through 10-fold cross-validation), 50% of seroconversions were classified as at elevated risk. Because region was incorporated in the score, a higher proportion of persons in higher incidence regions (western Kenya and western Uganda) as compared to lower incidence regions (eastern Uganda) were classified as at elevated risk.

The resulting risk-score was used during population-based testing in this study to provide real-time classification of persons as at elevated risk. HIV-uninfected individuals whose risk-score was greater than or equal to the cutoff were identified as at elevated risk and received enhanced counseling on PrEP. This risk-score has since been updated and evaluated using testing data from year three of the SEARCH test-and-treat trial;[3] results and additional methodological details are described in Balzer et al.[5]

References:

1. Koss CA, Ayieko J, Mwangwa F, et al. Early adopters of Human Immunodeficiency Virus preexposure prophylaxis in a population-based combination prevention study in rural Kenya and Uganda. *Clin Infect Dis* 2018; **67**(12): 1853-60.
2. Zheng W, Balzer L, van der Laan M, Petersen M, SEARCH Collaboration. Constrained binary classification using ensemble learning: an application to cost-efficient targeted PrEP strategies. *Stat Med* 2018; **37**(2): 261-79.
3. Havlir DV, Balzer LB, Charlebois ED, et al. HIV testing and treatment with the use of a community health approach in rural Africa. *N Engl J Med* 2019; **381**(3): 219-29.
4. van der Laan M, Polley EC, Hubbard AE. Super Learner. *Statistical Applications in Genetics and Molecular Biology* 2007; **6**(1).
5. Balzer LB, Havlir DV, Kamya MR, et al. Machine learning to identify persons at high-risk of HIV acquisition in rural Kenya and Uganda. *Clin Infect Dis* 2019.

Supplemental Table 1. Overlap between HIV risk categories (serodifferent partnership, risk score, and self-identified HIV risk) among 12,935 HIV-uninfected adult residents assessed to be at elevated HIV risk

		Serodifferent partnership[a] (N = 1,353)	Risk score (N = 7,256)	Self-identified HIV risk (N = 7,581)
**Self-identified HIV risk**	Yes	820 (61%)	2,316 (32%)	NA
	No	533 (39%)	4,940 (68%)	NA
**Risk score**	Yes	318 (24%)	NA	2,316 (31%)
	No	1,035 (76%)	NA	5,265 (69%)
**Serodifferent partnership**	Yes	NA	318 (4%)	820 (11%)
	No	NA	6,938 (96%)	6,761 (89%)

NA: not applicable.

a. Risk score was designed to identify persons at risk assuming all persons in serodifferent partnerships would be identified by other means and was thus trained to predict risk among persons not known to be in serodifferent relationships (rather than to predict serodiscordance).

Supplemental Table 2. Characteristics of HIV-uninfected men and women who initiated PrEP within 90 days in 16 communities in rural Kenya and Uganda

		**Men (n=1,733)**	**Women (n=1,756)**
**Age**, years	15-24	532 (31%)	446 (25%)
	25-34	544 (31%)	653 (37%)
	35-44	325 (19%)	427 (24%)
	45-54	198 (11%)	195 (11%)
	≥55	134 (8%)	35 (2%)
**Educational attainment**[a]	Less than primary	78 (5%)	146 (8%)
	Primary school	1,065 (61%)	1,306 (74%)
	Any secondary school/above	589 (34%)	301 (17%)
**Occupation**[b]	Farmer	737 (43%)	1,058 (60%)
	Student	121 (7%)	49 (3%)
	Fishing/bar/transportation	409 (24%)	139 (8%)
	Other informal sector	321 (19%)	322 (18%)
	Other formal sector	80 (5%)	69 (4%)
	Unemployed or disabled	51 (3%)	113 (6%)
	Other/unknown	13 (0·8%)	3 (0·2%)
**Marital status**[c]	Not married	559 (32%)	162 (9%)
	Married - monogamous	844 (49%)	944 (54%)
	Married - polygamous	225 (13%)	467 (27%)
	Divorced/separated/widowed	104 (6%)	181 (10%)
**Circumcised**[d]	Medical	513 (30%)	N/A
	Traditional	317 (18%)	N/A
	Uncircumcised	898 (52%)	N/A
**Alcohol use**[e]	None	1,176 (68%)	1,647 (94%)
	1-7 days/month	195 (11%)	62 (4%)
	>7 days/month	361 (21%)	47 (3%)
**Mobility**[f]	Yes	122 (7%)	70 (4%)
**Region**	Kenya	749 (43%)	793 (45%)
	Uganda East	457 (26%)	473 (27%)
	Uganda West	527 (30%)	490 (28%)

Data are number (%).
a. Missing data for 4 residents (0·11%).
b. Occupation: Other formal sector: teaching, government, military, health care, or factory. Other informal sector: shopkeeper, market vendor, hotel worker, homemaker, household worker, miner, or construction. Missing data for 4 residents (0·11%).
c. Missing data for 3 residents (0·09%).
d. Missing data for 5 residents (0·29%).
e. Missing data for 1 resident (0·03%).
f. Mobility: migration out of community for at least one month or moved residence within past 12 months. Missing data for 8 residents (0·23%).

Supplemental Table 3. PrEP uptake by population subgroups of 12,935 HIV-uninfected adult residents assessed to be at elevated HIV risk

	Uptake, n/N (%)
**Fishing/bar/transport workers**	548/2,349 (23%)
**Young women (15-24 years)**	446/2,376 (19%)
**Young men (15-24 years)**	532/2,424 (22%)
**Mobile individuals[a]**	192/1,131 (17%)
**Persons in serodifferent partnerships**	603/1,353 (45%)
Women in serodifferent partnerships	368/750 (49%)
Men in serodifferent partnerships	235/603 (39%)
**Persons identified by risk score**	1,226/7,256 (17%)
**Persons with self-identified HIV risk**	3,374/7,581 (45%)

a. Mobility: migration out of community for at least one month or moved residence within past 12 months

Supplemental Table 4. Factors associated with high adherence to PrEP (7 doses/week based on tenofovir hair concentrations) at week 4 among PrEP initiators self-reporting current risk in 16 communities in rural Kenya and Uganda[a]

		Odds ratio (95% CI)	p-value	Adjusted odds ratio (95% CI)	p-value
**Sex**	Male	1		1	
	Female	0·34 (0·11-1·01)	0·051	0·33 (0·13-0·84)	0·017
**Age**, years	15-24	0·33 (0·13-0·79)	0·013	0·24 (0·08-0·76)	0·015
	25-34	0·56 (0·25-1·28)	0·17	0·52 (0·21-1·24)	0·14
	35-44	1		1	
	≥45	1·68 (0·58-4·87)	0·34	1·12 (0·48-2·64)	0·79
**Serodifferent partnership**	No/unknown	1		1	
	Yes	3·02 (1·14-7·99)	0·026	2·64 (1·18-5·89)	0·018
**Occupation[b]**	Student/other formal sector occupation	1		1	
	Fishing/bar/transport	0·43 (0·10-1·77)	0·24	0·25 (0·45-1·38)	0·11
	Farming/other informal sector occup.	0·33 (0·08-1·25)	0·10	0·23 (0·05-0·98)	0·047
	Unemployed/disabled/other	1·14 (0·23-5·56)	0·87	0·79 (0·13-4·78)	0·79
**Mobility[b]**	No	1		1	
	Yes	1·92 (0·71-5·18)	0·20	1·83 (0·46-7·37)	0·39

a. Of 3,466 PrEP enrollees alive and not withdrawn at week 4, a total of 2,215 attended a week 4 visit, of whom 1,699 reported self-assessed risk of HIV and 1,230 self-reported adherence to PrEP. This analysis includes 635 participants seen at week 4 who reported self-assessed risk of HIV: 469 non-adherent to PrEP by self-report and 166 who were sampled for analysis of tenofovir hair concentrations from among 1,230 who self-reported adherence to PrEP. Odds ratios, with and without adjustment for other covariates, were estimated using mixed effects logistic regression with community as random effect and variances adjusted for clustering at community level, and using inverse sampling weights to account for sampling of tenofovir levels among persons with self-reported adherence. Specifically, we used inverse sampling weights for the 166 participants with tenofovir concentration data and assigned a weight of 1 for persons who self-reported risk and non-adherence.
b. Occupation: other formal sector: teaching, government, military, health care, or factory. Other informal sector: shopkeeper, market vendor, hotel worker, homemaker, household worker, miner, or construction.
c. Mobility: migration out of community for at least one month or moved residence within past 12 months.

Supplemental Table 5. Factors associated with adherence to PrEP (tenofovir hair concentrations measured as a continuous variable) at week 4 among PrEP initiators self-reporting current risk in 16 communities in rural Kenya and Uganda[a]

		**Coef. (95% CI)**	**p-value**	**Adjusted Coef. (95% CI)**	**p-value**
**Sex**	Male	1		1	
	Female	-0·19 (-0·59, 0·21)	0·32	-0·21 (-0·51, 0·093)	0·159
**Age**, years	15-24	-0·56 (-0·98, -0·13)	0·013	-0·50 (-1·07, 0·067)	0·080
	25-34	-0·46 (-0·87, -0·046)	0·032	-0·46 (-0·85, -0·078)	0·022
	35-44	1		1	
	≥45	0·18 (-0·31, 0·67)	0·45	0·024 (-0·36, 0·41)	0·90
**Serodifferent partnership**	No/unknown	1		1	
	Yes	0·65 (0·24, 1·06)	0·004	0·54 (0·07, 1·02)	0·027
**Occupation**	Student/other formal sector occupation	1		1	
	Fishing/bar/transport	0·43 (0·10, 1·77)	0·24	-0·47 (-1·16, 0·23)	0·18
	Farming/other informal sector occup.	0·33 (0·09, 1·26)	0·10	-0·058 (-0·67, 0·56)	0·84
	Unemployed/disabled/other	1·14 (0·23, 5·56)	0·87	0·12 (-0·76, 1·00)	0·78
**Mobility**	No	1		1	
	Yes	1·92 (0·71-5·18)	0·20	0·25 (-0·43, 0·92)	0·45

a. Of 3,466 PrEP enrollees alive and not withdrawn at week 4, a total of 2,215 attended a week 4 visit, of whom 1,699 reported self-assessed risk of HIV and 1,230 self-reported adherence to PrEP. This analysis includes 635 participants seen at week 4 who reported self-assessed risk of HIV: 469 non-adherent to PrEP by self-report and 166 who were sampled for analysis of tenofovir hair concentrations from among 1,230 who self-reported adherence to PrEP. Analyses were performed using linear regression. Inverse sampling weights were used to account for sampling of tenofovir levels among persons with self-reported adherence. Specifically, we used inverse sampling weights for the 166 participants with tenofovir concentration data and assigned a weight of 1 for persons who self-reported risk and non-adherence.
b. Occupation: other formal sector: teaching, government, military, health care, or factory. Other informal sector: shopkeeper, market vendor, hotel worker, homemaker, household worker, miner, or construction.
c. Mobility: migration out of community for at least one month or moved residence within past 12 months.

Supplemental Table 6. Grade 3, 4, and serious adverse events among 3,489 PrEP participants

	Description of adverse event	Number of events
**Grade 5 (death)**	Complications of alcohol use	1
	Diabetic ketoacidosis	1
	Drowning	1
	Injuries following motor vehicle accident	1
	Murder	1
	Tuberculosis	1
	Unknown cause	1
**Grade 4**	Anemia	2
	Pre-eclampsia	1
	Ruptured ectopic pregnancy	1
	Soft tissue injury	1
	Suicide attempt by poisoning	1
**Grade ≤3**	Abdominal Pain	4
	Anemia	1
	Back pain	1
	Bilirubin elevation	1
	Creatinine elevation	1
	Dizziness	2
	Fatigue	1
	Fractures of upper and lower limbs following motor vehicle accident	1
	Headache	1
	Injury to lower limbs	1
	Pulmonary tuberculosis	2
	Spontaneous abortion	5
	Stillbirth	2
	Typhoid fever	1
	Uterine bleeding	1
	Uterine fibroids	1
